# Wood Fires Again

**Published:** 1871-12

**Authors:** 


### WOOD FIRES AGAIN.

The men and women of the present generation, who were boys and girls forty or fifty years ago, can never forget the old-fashioned fire-places, with their shovel and tongs, the andirons, the screen, and the dear old hearthstones; with pussy cat on one side, the dog on the other; father and mother in front, and Tom and Bet and Bill tumbling over the floor, or teasing Nellie and Jenny out of their lives; and then the apples roasting all around in a circle; the flickering of the flames, the shadows dancing on the wall; the joke, the laugh, the pleasantry. But mother and father are gone now! Pretty pussy cat purrs no more in our lap, the dog is dead, the fire has gone out, and there has been a woful breaking up generally. The mason has come and bricked up the fire-place; the dog-irons have been stowed away in the garret for thirty years or more; we saw them last week, all covered with dust and cobwebs and canker spots; how they hurried us back to Days Lang Syne. But lo and behold, the open fire-place is coming back again; the brass andirons, the shovel and tongs, the logs all aglow, the flickering flames, and the dancing shadows on the wall; only father and mother are not there; for on June the sixth, 1871, Thomas Dixon & Sons, of Philadelphia, prepared an imitation of a wood fire, which for economy and beauty and cheeriness and health is one of the most important domestic inventions of the times; and we hope the day is not far distant when one at least will have a place in every family in the nation. It is so arranged that it can be supplied with gas, and being lighted, it cannot be discerned from a common wood fire of the olden time. It is a positive godsend to all gloomy, cheerless, chilling parlors, reception and family sitting rooms. Everybody of any observation knows what a thrill of pleasure goes through the frame the instant of entering an apartment of a cold day, on seeing a brisk flame dancing in the fire-place; everybody knows too, what a dreary thing it is to go into a modern parlor of a winter's day, and see no sign of light or life; all is still and sombre as the night itself, and there is a heavy closeness in the air you breathe, which takes all the spirit and animation out of you in an instant, and you are only too glad when the visit is ended. The patent is called "Gas-log," and costs from twelve to twenty-five dollars according to the size of the fire-place; the cost of running it averages ten cents an hour or less, with gas at three dollars a thousand cubic feet. The logs look like hickory wood, and last for a lifetime; the fire is kindled as instantly as a lamp is lighted, without dust or smoke or odor of gas, as all go up the chimney. It can be lighted or extinguished in an instant, and is an admirable arrangement for ventilating the chambers of the sick at night, when it is too cold to hoist the windows; for when there is even a light fire on the hearth, the air coming in through the cracks and crevices of doors and windows, creates a current up the chimney, which carries away the impure atmosphere of the apartment, and keeps it fresh and invigorating.

